# A novel method to measure hairiness in bees and other insect pollinators

**DOI:** 10.1002/ece3.6112

**Published:** 2020-02-27

**Authors:** Laura Roquer‐Beni, Anselm Rodrigo, Xavier Arnan, Alexandra‐Maria Klein, Felix Fornoff, Virginie Boreux, Jordi Bosch

**Affiliations:** ^1^ CREAF Universitat Autònoma de Barcelona Cerdanyola del Vallès Catalunya Spain; ^2^ Nature Conservation and Landscape Ecology University of Freiburg Freiburg Germany

**Keywords:** functional diversity, functional trait, pilosity, pollinating efficiency, protocol, thermoregulation

## Abstract

Hairiness is a salient trait of insect pollinators that has been linked to thermoregulation, pollen uptake and transportation, and pollination success. Despite its potential importance in pollination ecology, hairiness is rarely included in pollinator trait analyses. This is likely due to the lack of standardized and efficient methods to measure hairiness. We describe a novel methodology that uses a stereomicroscope equipped with a live measurement module software to quantitatively measure two components of hairiness: hair density and hair length. We took measures of the two hairiness components in 109 insect pollinator species (including 52 bee species). We analyzed the relationship between hair density and length and between these two components and body size. We combined hair density and length measures to calculate a hairiness index and tested whether hairiness differed between major pollinator groups and bee genera. Body size was strongly and positively correlated to hair length and weakly and negatively correlated to hair density. The correlation between the two hairiness components was weak and negative. According to our hairiness index, butterflies and moths were the hairiest pollinator group, followed by bees, hoverflies, beetles, and other flies. Among bees, bumblebees (*Bombus*) and mason bees (*Osmia*) were the hairiest taxa, followed by digger bees (Anthophorinae), sand bees (*Andrena*), and sweat bees (Halictini). Our methodology provides an effective and standardized measure of the two components of hairiness (hair density and length), thus allowing for a meaningful interpretation of hairiness. We provide a detailed protocol of our methodology, which we hope will contribute to improve our understanding of pollination effectiveness, thermal biology, and responses to climate change in insects.

## Graphical abstract

Hairiness is a fundamental functional trait in pollinator ecology related to thermoregulation and pollination effectiveness. Surprisingly, there is a lack of standard methods to measure this trait. We provide an effective method to quantitatively measure hairiness to contribute to filling an important gap in pollinator data bases and to improve our understanding of the ecology and evolution of a threatened group of organisms that provide an essential ecosystem service.

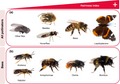

## 1 INTRODUCTION

Functional traits are morphological, physiological, or phenological characteristics measurable at the individual level, which are believed to influence the fitness of an organism, to be a response of the organism to environmental changes or to reflect the effect of the organism on ecosystem function (Violle et al., 2007). A growing number of studies are exploring the relationship between functional trait diversity, environmental change and species composition, and emphasizing the importance of functional diversity in ecosystem processes (e.g., Arnan, Cerdá, Rodrigo, & Retana, 2013; Elmqvist et al., 2003; Hooper et al., 2005; Petchey & Gaston, 2006; Suding et al., 2008). Trait‐based studies were mostly pioneered by plant ecologists, and extensive trait data bases are available for many plant taxa and communities (e.g., Díaz et al., 2007; Lavorel & Garnier, 2002; McIntyre, Lavorel, Landsberg, & Forbes, 1999). Compared with plants, we know much less about animal functional diversity, especially of terrestrial invertebrate communities (Moretti et al., 2017; Parr et al., 2017). This is partly caused by the lack of standardized protocols to measure functional traits in terrestrial invertebrates (Didham, Leather, & Basset, 2016; Moretti et al., 2017). Consequently, some important traits are often not measured or measured in ways that are not comparable across studies, rendering databases insufficient, non‐uniform, or taxa‐limited.

Pollinators play a key role in the functioning of terrestrial ecosystems and provide an essential ecosystem service in terms of crop pollination (Klein et al., 2007). However, several studies in Europe and North America have shown that pollinator diversity is declining (Bartomeus & Winfree, 2013; Biesmeijer, 2006; Colla, Gadallah, Richardson, Wagner, & Gall, 2012; Powney et al., 2019). Within this context, functional traits are increasingly being incorporated in pollinator studies. Various studies have established links between environmental changes and species susceptibility (Murray, Kuhlmann, & Potts, 2009; Roulston & Goodell, 2011) and between biodiversity and ecosystem functioning (Fontaine, Dajoz, Meriguet, & Loreau, 2006; Gagic et al., 2015). Commonly used functional traits in pollinator ecology studies include body size, mouthpart length, sociality, trophic specialization (lecty), voltinism, flight period, and nesting habits (e.g., Aguirre‐Gutiérrez et al., 2016; Coutinho, Garibaldi, & Viana, 2018; De Palma et al., 2015; Woodcock et al., 2019).

One particularly important trait in pollinator insects is hairiness (pilosity). Hairiness creates an insulation layer that mitigates convective loss of heat generated by the vibration of thoracic muscles, thus playing an essential role in thermoregulation (Heinrich, 1993; May, 1979). Some studies have found differences in hair length between bees from different climates (Peat, Darvill, Ellis, & Goulson, 2005) and along elevation gradients (Peters, Peisker, Steffan‐Dewenter, & Hoiss, 2016), suggesting that hairiness could act as a response trait to climatic changes. Hairiness can also be considered an effect trait involved in pollen collection and transfer (Amador et al., 2017; Müller, 1995; Thorp, 2000), potentially affecting pollination effectiveness (Phillips, Williams, Osborne, & Shaw, 2018; Stavert et al., 2016; Woodcock et al., 2019).

Notwithstanding the importance of hairiness in pollinator ecology, information on how to measure this trait is scarce and inconsistent across studies (Moretti et al., 2017). As a result, hairiness data are mostly lacking in pollinator data bases and, when available, are not comparable across studies. Some studies use thorax hair length as a measure of hairiness (Peat et al., 2005; Peters et al., 2016). Others use the percentage of body surface covered by hair (Kühsel, 2015; Phillips et al., 2018). However, these measures do not account for the two components of hairiness (hair length and hair density; Moretti et al., 2017). Other studies do consider both components, but use a semi‐quantitative scale (Woodcock et al., 2019). Finally, Stavert and collaborators (2016) proposed an innovative method that uses a measure of entropy obtained from images of the insect's body surface as a proxy for hairiness. However, we could not apply this method to pollinators with shiny cuticles, which yielded high levels of entropy due to light reflection.

The aim of our study was to develop a method to quantitatively measure hairiness in insect pollinators. We describe procedures to measure hair density and hair length and propose a simple hairiness index integrating both components. These procedures are then synthesized in a standardized protocol. We apply this protocol to three different body parts of 109 insect pollinator species and show that our methodology discriminates pollinator groups and bee genera in terms of hairiness. Finally, because body size is another functional trait that has been related to both thermoregulation (Heinrich, 1993; Stone & Willmer, 1989) and pollination effectiveness (Jauker, Speckmann, & Wolters, 2016; Kandori, 2002; Willmer & Finlayson, 2014), we explore the relationship between hair density, hair length, and body size.

## 2 MATERIALS AND METHODS

### 2.1 Insect specimens

We used a collection of pollinator insects from Sweden, Germany, and Spain composed of 109 species including Anthophila (bees; 52 species), Syrphidae (hoverflies, 27), Bombyliidae (bee‐flies, 2), other flies (9), Coleoptera (beetles, 9), Lepidoptera (butterflies and moths, 5), Vespidae (wasps, 3), and Symphyta (saw‐flies, 2). Bees comprised the following genera: *Andrena* (20 species), *Bombus* (10), *Lasioglossum* (8), *Halictus* (3), *Osmia* (5), *Apis* (1), *Anthophora* (1), *Eucera* (2), *Xylocopa* (1), and *Nomada* (1). For the analyses, *Lasioglossum* and *Halictus* species (Halictini) were grouped together, as well as *Anthophora, Eucera, and Xylocopa* species (Anthophorinae) (Table S1). Because bees show marked sexual dimorphism, we only worked with females.

### 2.2 Body size

We used body length as an estimator of body size (mean ± SE sample size = 5.44 ± 0.39 specimens per pollinator species). For bees, in addition to body length, we measured intertegular span (hereafter ITS) using a stereomicroscope (mean ± SE sample size = 6.62 ± 0.68 specimens per species). ITS is the most commonly used estimator of body mass in bee studies (Cane, 1987; Cariveau et al., 2016; Kendall et al., 2019; Osorio‐Canadas et al., 2016). For this reason, we used ITS in the analyses involving only bees and body length in the analyses involving all pollinators. ITS and body length were highly correlated in bees (Spearman *ρ* = 0.87; *p* < .001).

### 2.3 Hairiness

**Figure 1:**
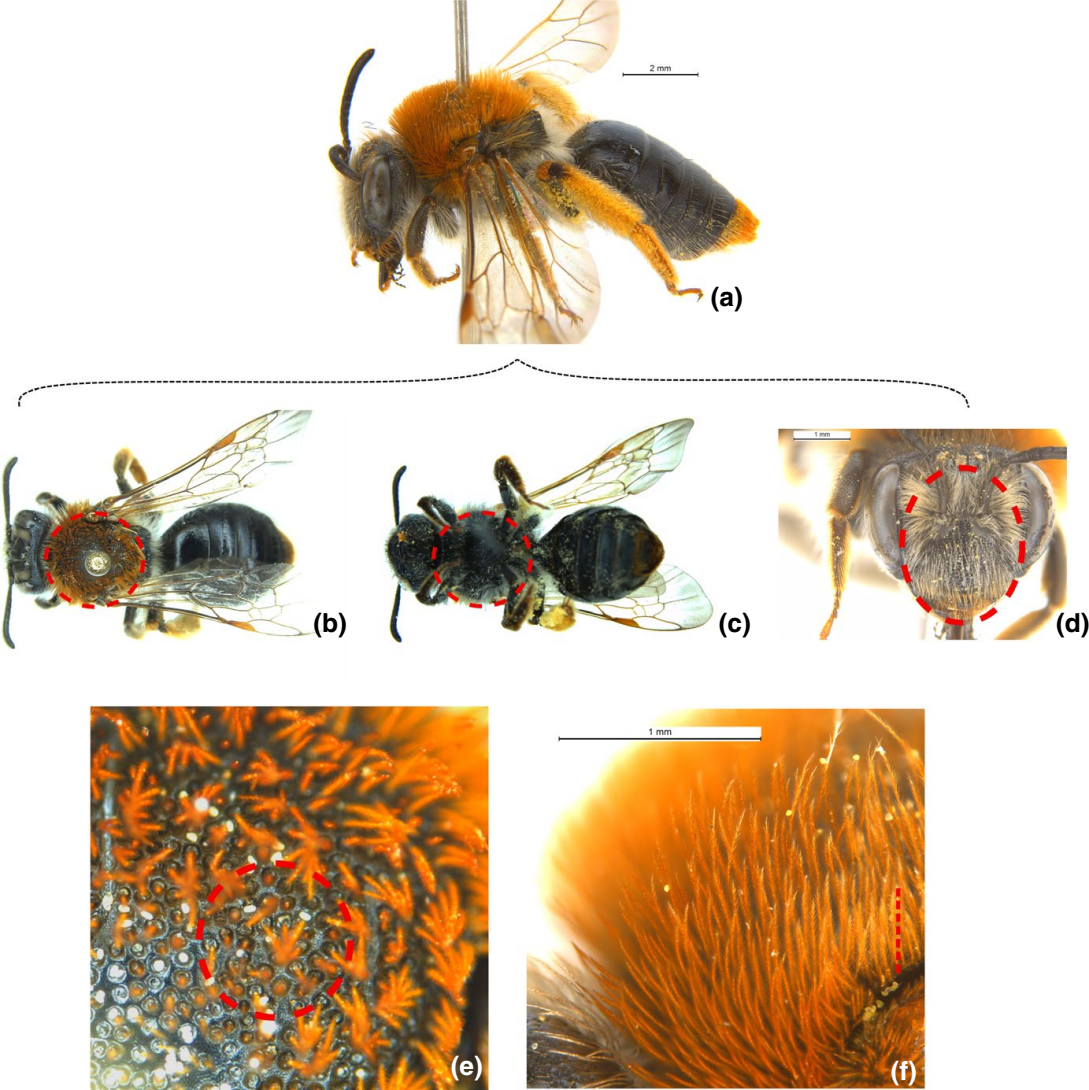
Pinned *Andrena haemorrhoa* female (a), body parts in which hairiness was measured (b: dorsal surface of the mesothorax, c: ventral surface of the mesothorax, d: face), and close‐up images of the dorsal mesothorax showing measurements of hair density (e) and hair length (f)

The two components of hairiness (hair density and hair length) were measured in three body parts: the dorsal surface of the mesothorax, the ventral surface of the thorax, and the face (Figure 1a‐d). We selected these body parts because the flight muscles involved in endogenous heat production are located in the thorax (Heinrich, 1993) and because the thorax and the face act as surfaces of pollen exchange in the pollination of many flower species (Willmer, 2011). We measured a mean of three specimens per species (mean ± SE = 2.96 ± 0.11). Measurements were taken with the stereomicroscope LEICA M165C equipped with a LAS live measurement module software (Leica Microsystems). This module allows taking length and surface measurements on live images in real units (Figure 1e,f).

#### 2.3.1 Hair density (number of hairs/mm^2^)

In each of the three above‐mentioned body parts, we selected 3 representative areas of approximately 0.1 mm^2^ and counted the number of hairs in each area. In some cases, notably in species with high hair density and in specimens in which hairs formed clumps due to manipulation during capture and/or preservation, it was easier to count hairs at their insertion points, usually signaled by a micropore on the cuticle (Figure 1e, see Appendix 1 for details). Counting micropores has the added advantage that can be applied to specimens that have lost hairs (e.g., due to aging; Bosch & Vicens, 2006; Southwick, 1985; or to poor manipulation) to obtain a measure of original hair cover.

The results of these three measurements were used to calculate a mean hair density for each body part. In some species, hairiness patterns were clearly not uniform within a body part (notably in the face). In these cases (11.3% of the 327 species/body parts we measured), we sampled approximately 0.1 mm^2^ of the area occupied by each hairiness pattern separately, and the overall hair density mean was weighted by the area occupied by each hairiness pattern.

#### 2.3.2 Hair length (mm)

The length of 8–9 hairs of each body part was measured using the length measuring tool of the software (Figure 1f). Again, in body parts with clearly distinct hairiness patterns, hair length of 8–9 hairs was measured separately for each part and the overall mean hair length was weighted by the surface occupied by each hairiness pattern.

The time spent measuring hairiness (hair length + hair density in three 0.1 mm^2^ areas of the three body parts) was about 15 min per specimen. A detailed protocol describing our method can be found in Appendix 1.

### 2.4 Data analysis

All analyses were conducted in R v.3.3.2 (R Core Team, 2016), first with all pollinator species, and then with bees only.

#### 2.4.1 Relationships between hair density, hair length, and body size

For each body part separately, we tested whether hair density and hair length were correlated. We also tested the correlation of each of these two hairiness components with body size. Because hair density and length were only weakly correlated (see results), we calculated, for each body part, a hairiness index (hair density × hair length). Finally, we examined whether the hairiness components and the hairiness index of the three different body parts were correlated. We used either the Pearson or Spearman correlation depending on data distribution.

#### 2.4.2 Hairiness comparisons among pollinator groups and bee taxa

We calculated the coefficients of variation (*SD*/mean × 100) of the two hairiness components within and between species, separately for each body part. Because our method accounts for hair loss and since we only measured female specimens, we expected greater variability between than within species.

We explored whether hair density, hair length, and hairiness index differed between pollinator groups and bee taxa using one‐way ANOVA and Kruskal‐Wallis tests (depending on data distribution), followed by post hoc tests for multiple comparisons (Tukey's and Dunn's tests, respectively). We analyzed each hairiness component of each body part separately. Log (X + 1) and square‐root transformations were applied to improve normality and homoscedasticity of model residuals if needed. Pollinator groups and bee taxa with three or fewer species (bee‐flies, saw‐flies, wasps, *Apis*, *Nomada*) were excluded from the analyses but their values are provided in the figures.

## 3 RESULTS

### 3.1 Hairiness components: hair density and hair length

Hair density of all pollinators ranged from 0 to 5,797.6 hairs/mm^2^ (mean ± SE = 428.0 ± 32.3) and that of bees from 63.5 to 1,052.2 (mean ± SE = 333.4 ± 11.6). Hair length of all pollinators ranged from 0.01 to 1.91 mm (mean ± SE = 0.48 ± 0.02) and that of bees from 0.09 to 1.58 mm (mean ± SE = 0.62 ± 0.03).

**Figure 2:**
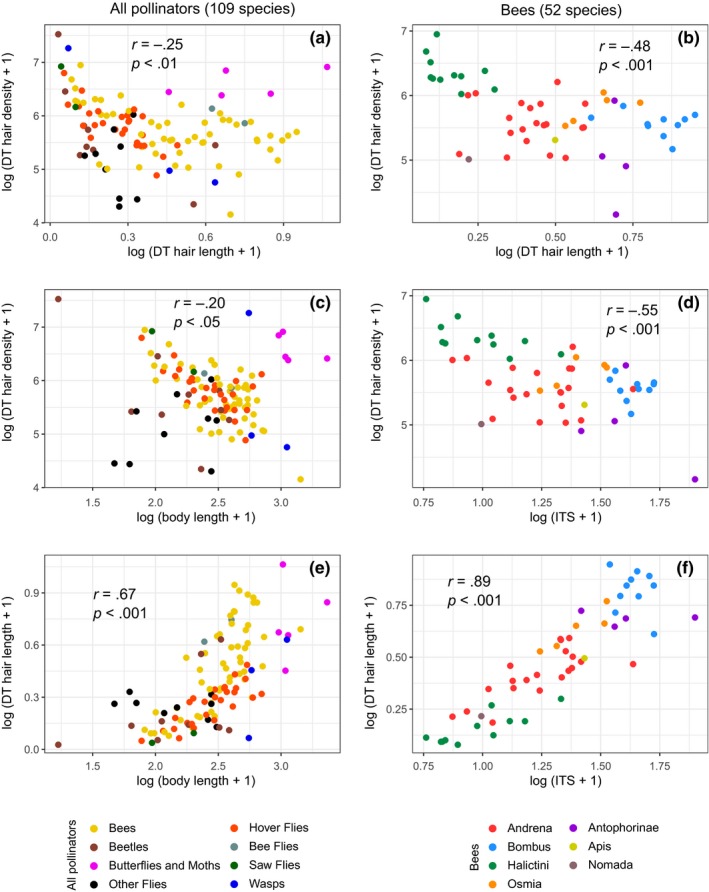
Scatter plots showing the relationship between hair density and hair length (a and b), between hair density and body size (c and d) and between hair length and body size (e and f); of the dorsal surface of the mesothorax for all pollinators (a, c, and e; 109 species) and for bees only (b, d, and f; 52 species). Each point corresponds to one species. See Table S2 for results of the ventral surface of the mesothorax and the face. DT, dorsal surface of the mesothorax; ITS, intertegular span

Dorsal thorax hair density and length were weakly and negatively correlated (all pollinators: *r *= −.25, *p* < .01; bees: *r *= −.48; *p* < .001; Figure 2a,b). Ventral thorax and face hair density and length were also weakly and negatively correlated for bees (*r *= −.38 and −.39, respectively; *p* < .01), but not for all pollinators (*p* > .08).

### 3.2 Relationship between hairiness components and body size

Dorsal thorax hair density and body size were negatively correlated, weakly for all pollinators (*r *= −.20, *p* < .05, Figure 2c) and moderately for bees (*r *= −.55, *p* < .001, Figure 2d). The analysis of ventral thorax and face hairiness yielded similar results (Table S2). Conversely, hair length and body size were positively and strongly correlated in all three body parts (all pollinators, *r* = .67–.70, *p* < .001, Figure 2e; bees, *r* = .89–.93, *p* < .001, Figure 2f).

### 3.3 Hairiness comparisons among body parts

Hairiness was positively correlated across body parts (all *p* < .001, Table S3). Correlation coefficients were higher for hair length (all pollinators: *r* = .91–.95; bees: *r* = .96) than for hair density (all pollinators: *ρ* = 0.47–0.67; bees: *r* = .61–.74). The hairiness index was also strongly correlated across body parts (all pollinators: *r* = .78–.88; bees: *r* = .77–.85).

### 3.4 Differences in hairiness across pollinator groups and bee taxa

Since both hairiness components and the hairiness index were correlated among body parts, hereafter, we only show results of the dorsal region of the thorax (the analysis of the ventral region of the thorax and the face yielded similar results; see Tables S4 and S5; Figures S3 and S4).

The coefficient of variation of dorsal thorax hairiness components was much higher between species (all pollinators: 73.0%–76.7%; bees: 53.4%–60.7%) than within species (all pollinators: 17.1%–18.7%; bees: 17.0%–18.3%; Table S4).

#### 3.4.1 All pollinators

**Figure 3:**
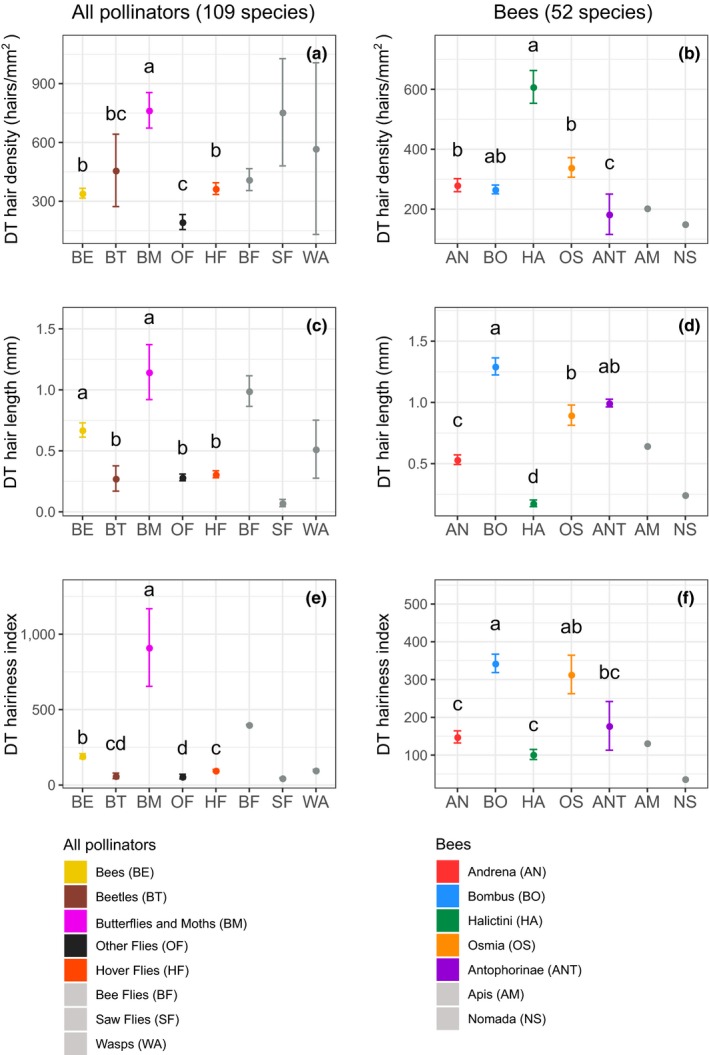
Mean ± SE hair density (a and b), hair length (c and d), and hairiness index (e and f) of the dorsal surface of the mesothorax (DT) of various pollinator groups and bee taxa. Different letters indicate significant differences among groups (post hoc Tukey's tests, *p* < .05). Groups with fewer than three species (in gray) were not included in the analyses

**Figure 4:**
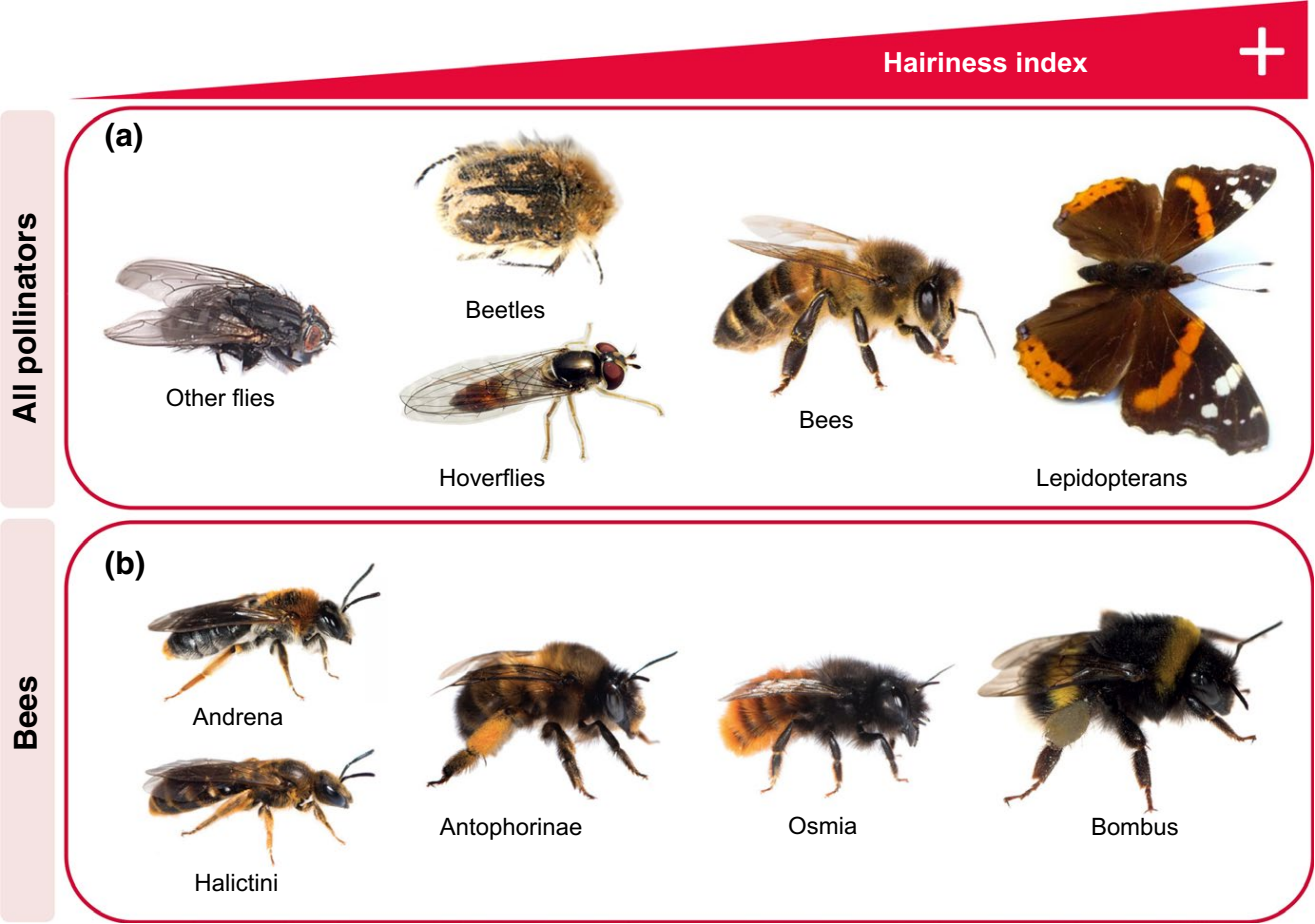
Pollinator groups (a) and bee taxa (b) ordered by increasing hairiness from left to right. (Photograph credits Nicolas J. Vereecken [all bees], Adrià Miralles [hoverflies] and Laura Roquer‐Beni [other flies, beetles and lepidopterans]. All images used with permission.)

We found clear differences among pollinator groups in the two hairiness components and the hairiness index (Figures 3 and 4a). Butterflies and moths were the group with the highest hair density, followed by bees, hoverflies, and beetles; other flies were the group with the lowest hair density (ANOVA, *F*_4,97_ = 6.9, *p* < .001, Figure 3a). Butterflies and moths and bees had longer hair than any of the other pollinator groups (*F*_4,97_ = 11.3, *p* < .001, Figure 3c). According to the hairiness index, butterflies and moths were the hairiest group followed by bees, hoverflies, beetles, and other flies (*F*_4,97_ = 27.2, *p* < .001, Figure 3e).

#### 3.4.2 Bees

Our measures of hairiness also yielded clear differences among bee taxa (Figures 3 and 4b). Halictini had the highest hair density, followed by *Bombus, Osmia, and Andrena*, and Anthophorinae had the lowest density (*F*_4,45_ = 16.25, *p* < .001, Figure 3b). This pattern changed completely for hair length. *Bombus* and Anthophorinae had the longest hair followed by *Osmia* and *Andrena*, and Halictini had the shortest hair (*F*_4,45_ = 67.9, *p* < .001, Figure 3d). According to the hairiness index, the hairiest taxa were *Bombus* and *Osmia*, followed by Anthophorinae, *Andrena* and Halictini (*F*_4,45_ = 13.5, *p* < .001, Figure 3f).

## 4 DISCUSSION

The aim of our study was to establish a standard practical procedure to quantitatively measure the two components of hairiness and to promote the use of this important trait in pollinator studies.

### 4.1 Advantages of the method

The methodology we describe has several advantages. First, it provides a quantitative measure of hair density and length. Second, it is a non‐invasive methodology; specimens remain undamaged. Therefore, our methodology can be applied not only to dead specimens, but also to live (anesthesized) insects. Third, it can be applied to specimens in which the hair cover has been altered due to manipulation. Tufts of clumped hair are common in specimens that have been exposed to high concentrations of ethyl acetate in killing jars and in specimens that have been kept in water or alcohol (e.g. specimens obtained from pan of malaise traps). Fourth, it accounts for hair loss due to aging or poor preservation. Certain pollinator groups, notably bee‐flies, tend to lose hairs during capture and manipulation. Thus, if needed, it is possible to obtain a measure of original hair density (as opposed to actual hair density). Fifth, measurements are taken directly from the specimen rather than from photographs. For this reason, our method is not affected by shininess, a common feature of the cuticle of many pollinator insects. Sixth, our method discriminates the two components of hairiness, thus allowing for a meaningful interpretation of the functional and evolutionary consequences of hairiness.

Although our method may appear to be time‐consuming, a trained person can process a specimen (8–9 measures of hair length + 3 measures of hair density in 3 body parts) in just 15 min. This amount of time can be reduced if, depending on the objectives of the study, fewer body parts are considered.

### 4.2 Relationship between hair density, hair length and body size

We found a negative (albeit weak) correlation between hair length and density for all three measured body parts in bees and for the dorsal region of the thorax in all pollinators. Accounting for the two components of hairiness would be redundant if these two variables were highly correlated. Some groups such as beetles and Halictini had very short hair but very high hair density (Figure 3). Other groups such as butterflies and moths and bees of the genera *Bombus* and *Osmia*, had long hair and high hair density.

Accounting for the two components of hairiness is also important because hair length and hair density may be differently related to body size. A positive relationship between hair length and body size is expected due to allometric and mechanical constraints (movement would be impaired in a small animal with long hair). Previous studies have found a positive relationship between hair length and body size at the intraspecific level in bumblebees (Goulson et al., 2002; Peat et al., 2005). Our results show that this relationship holds at the interspecific level and when pollinators from different orders are considered. Positive relationships between body size and length of various appendages are common in insects (proboscis: Cariveau et al., 2016; Kunte, 2007; legs: Kaspari & Weiser, 1999; Teuscher, Brändle, Traxel, & Brandl, 2009 and wings: Bosch & Vicens, 2002; Bullock, 1999).

The relationship between hair density and body size, on the other hand, is less straightforward. We cannot think of any a priori reason why hair density should differ between large and small animals. We found that the relationship between hair density and body size was weak and negative, especially in bees. Interestingly, studies on various groups of mammals have also found that small species tend to have denser (and shorter) fur (Sandel, 2013; Schwartz & Rosenblum, 1981; Steudel, Porter, & Sher, 1994). We suggest that the negative relationship between hair length and hair density, rather than indicative of a direct trade‐off, can be explained through the relationship between these two variables and body size. Given that small animals cannot have long hair due to the above‐mentioned mechanical constraints, the evolutionary pathway to achieve high levels of hairiness in small animals is through increased hair density.

### 4.3 Hairiness as an effect trait

Our methodology and our results have important implications for studies on pollination effectiveness. The ability to incorporate, transport, and deliver pollen is likely to be influenced by the two hairiness components. Longer hairs provide a greater surface for pollen grain adherence, and hair spacing (the inverse of hair density) may be important in relation to pollen grain size (Haider, Dorn, Sedivy, & Müller, 2014; Roberts & Vallespir, 1978), which shows great variability among plant taxa (Willmer, 2011).

A link between hairiness and pollination effectiveness has been found in some studies (Phillips et al., 2018; Stavert et al., 2016). Given the positive correlation between body size and hair length, studies exploring the relationship between hairiness and pollination effectiveness should account for body size, which, along with flower‐handling behavior and visit duration, has also been shown to affect pollination effectiveness (Jauker et al., 2016; Kandori, 2002; Phillips et al., 2018; Willmer & Finlayson, 2014). Accordingly, in pollination studies, hairiness measures should target the body parts involved in pollen transfer, which depend on flower morphology, pollinator body size and intra‐floral foraging behavior (Araujo, Medina, & Gimenes, 2018; Beattie, Breedlove, & Ehrlich, 1973; Bosch, 1992; Solís‐Montero & Vallejo‐Marín, 2017).

### 4.4 Hairiness as a response trait

Our methodology can also be important for studies on thermal biology and studies exploring the geographical distribution of pollinator communities and populations and their response to climate change. Some pollinators generate heat endogenously by contracting their flight muscles (Heinrich, 1993), and hairiness provides an insulation layer around the body surface that slows convective heat loss (May, 1979). As with pollination effectiveness, both components of hairiness (length and density) are likely to contribute to the creation and maintenance of this insulation layer and therefore to influence thermoregulation (Steudel et al., 1994; Wasserman & Nash, 1979). Consequently, we would expect pollinator species and populations to be hairier in colder climates. Again, given the correlation between hair length and body size, studies addressing the distribution of pollinators in relation to climate should account for body size. Body size is strongly related to the ability to generate heat and fly at low temperatures both at the intra‐ and interspecific levels (Bishop & Armbruster, 1999; Heinrich, 1993; Osorio‐Canadas et al., 2016; Stone, 1993; Stone & Willmer, 1989). Both body size and hair length of bumblebees have been shown to be greater in species from colder areas along latitudinal (Peat et al., 2005) and elevational gradients (Peters et al., 2016). Since most endogenous heat is produced by the flight muscles (Heinrich, 1993), measures of hairiness in thermal biology studies should mainly target the thorax, although other body parts (head, abdomen) have also been shown to be involved in heat loss (Cooper, 1985; Heinrich & Buchmann, 1986; Roberts & Harrison, 1998).

### 4.5 Hairiness in trait‐based studies

Functional diversity studies typically characterize species based on suites of traits. Ideally, these traits should be biologically meaningful, easy to measure and comparable across taxa. In principle, and until we have a better understanding of the mechanistic effects of hair length and hair density of different body regions on various ecological functions, we suggest keeping these two measures as separate traits in a multitrait space. Otherwise, if a single measure is desirable, they can be combined into a single trait (hairiness index).

### 4.6 Concluding remarks

We have developed a standardized procedure to measure hairiness and explored the relationships between hairiness components and between hairiness and body size. Overall, these relationships were similar when analyzing only bees and when analyzing all pollinators. Importantly, in addition to insect pollinators, our methodology can be applied to other groups of terrestrial arthropods and can be used to explore the relationships between hairiness and other ecological functions besides those discussed above. Hairiness has been shown to act as a physical and sensory barrier against predators and parasites in caterpillars (Castellanos, Barbosa, Zuria, Tammaru, & Christman, 2011; Lindstedt, Lindström, & Mappes, 2008; Sugiura & Yamazaki, 2014) and moths (Shen, Neil, Robert, Drinkwater, & Holderied, 2018). We hope our methodology will foster the inclusion of this important trait in insect data bases and will contribute to our understanding of the importance of hairiness in insect ecology.

## CONFLICT OF INTEREST

None declared.

## AUTHORS' CONTRIBUTIONS

LRB, JB, AR, and XA conceived the study; LRB conducted the measurements, analyzed the data, and led the preparation of the manuscript with the help from JB, AR, and XA. All authors provided insect specimens, contributed to the development of the methodology, and/or provided input during the preparation of the manuscript. All authors provided final approval for publication.

## Figures and Tables

**Figure A1:**
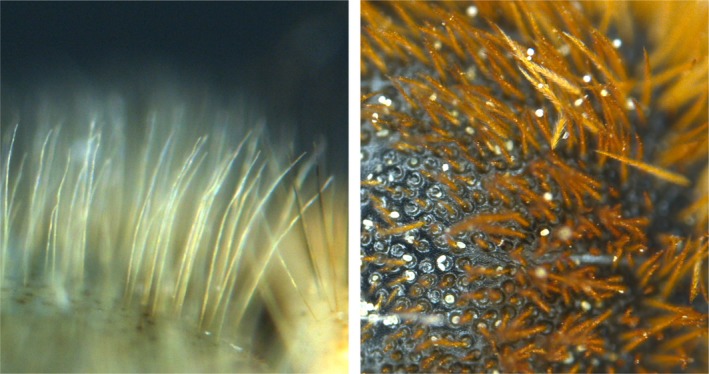
Hair micropores in the dorsal thorax region of *Scaeva albomaculata* (left) and of *Andrena haemorrhoa* (right)

**Figure A2:**
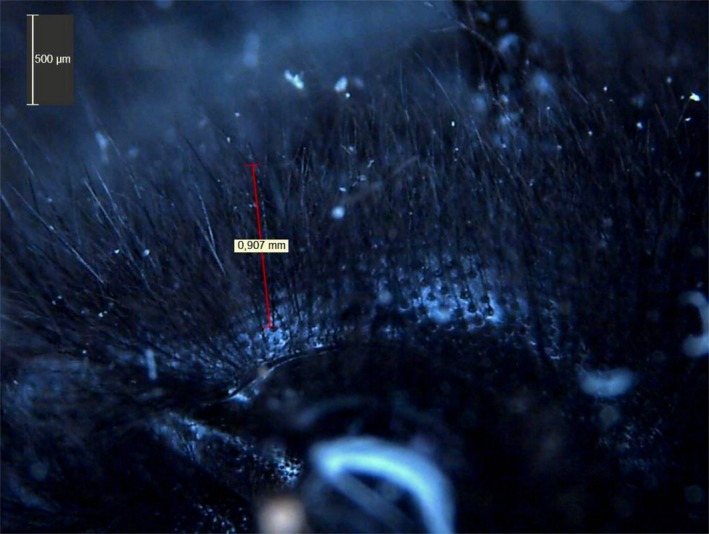
Measurement of hair length on the dorsal thorax region of *Xylocopa violacea*

## Data Availability

The data generated in this study are available at Dryad Digital Repository: https://doi.org/10.5061/dryad.3ffbg79f0

## Appendix 1

### PROTOCOL TO MEASURE HAIRINESS IN BEES AND OTHER INSECT POLLINATORS

This protocol follows the structure of the “Handbook of protocols for standardized measurement of terrestrial invertebrate functional traits” (Moretti et al., 2017). We focus on insect pollinators, but our methodology can also be applied to other groups of arthropods.

#### Definition and relevance

Insect hairiness (pilosity) is the collective presence of hairs, scales, seta or bristles growing from the cuticle (Moretti et al., 2017). Hairiness creates an insulation layer that mitigates the convective loss of heat generated by the vibration of thoracic muscles, thus playing an essential role in thermoregulation (Heinrich, 1993; May, 1979). Some studies have found a negative relationship between bee hair length and ambient temperature (Peat, Darvill, Ellis, & Goulson, 2005; Peters, Peisker, Steffan‐Dewenter, & Hoiss, 2016), suggesting that hairiness acts as a response trait to climate. Hairiness can also be considered an effect trait involved in pollen collection and transfer (Amador et al., 2017; Müller, 1995; Thorp, 2000), potentially affecting pollination effectiveness (Phillips, Williams, Osborne, & Shaw, 2018; Stavert et al., 2016; Woodcock et al., 2019). Hairiness may also be involved in antipredator strategies. In caterpillars and moths, hairs have been shown to provide mechanical and sensory defence against predators and parasites (Castellanos, Barbosa, Zuria, Tammaru, & Christman, 2011; Lindstedt, Lindström, & Mappes, 2008; Shen, Neil, Robert, Drinkwater, & Holderied, 2018; Sugiura & Yamazaki, 2014).

#### Technical support

Measurements should be taken with a stereomicroscope with a magnification range of 20–80× and a camera connected to a computer equipped with a software module that allows taking length and surface measurements directly on live images in real units. We used a LEICA M165C and the live measurement module from Leica Microsystems. Alternatively, other stereomicroscopes and software packages could be used (e.g., free software ImageJ). Measurements can also be taken from microscope pictures (instead of live images) but this is a more time‐consuming alternative. If real units are not provided by the software, then the level of magnification must be accounted for.

#### Pre‐treatment of the specimens

The method can only be applied to dry specimens. Apart from this, no special pre‐treatment is necessary. The method works well even with specimens that have lost hair due to aging or poor manipulation, as well as with specimens with clumps of hair (e.g., specimens initially kept in alcohol that have not been properly dried). The method can also be applied to anesthesized live specimens.

#### Body parts

Hairiness is best measured in body parts with flat surfaces, but the method can be applied to any body part. Hairiness may strongly differ among body parts. The target body part(s) should be decided based on the objectives of the study. A study on pollination effectiveness should target body parts directly involved with the uptake and transfer of pollen. A study on thermoregulation should emphasize body parts involved in heat generation (thorax) and dissipation (abdomen, head, appendages).

We found hairiness to be correlated across the face, the dorsal surface of the thorax and the ventral surface of the thorax.

#### Measurement of hairiness components

Hairiness can be decomposed in hair density and hair length (Moretti et al., 2017).

##### Hair density

Counting all hairs in an entire body part is unpractical. We found that averaging hair counts of three areas of ca. 0.1 mm^2^ each provides a good measure of hair density for a given body part. The area sampled (and if needed the area of the entire body part) can be measured with the appropriate tool in the software.

Counting hairs can be complicated when hair density is high and when hairs are long and form clumps due to manipulation during capture and/or preservation. In these cases, it is easier to count the micropores of the cuticle in which hairs are inserted (Figure A1). Because insect cuticles may display a large variety of microsculpture patterns, including several types of punctuations, it is very important to spend some time to identify the correct type of micropores before starting the counts. Counting micropores has the added advantage that it provides a measure of original hairiness even in specimens that have lost hair due to aging or poor manipulation (e.g., bee‐flies typically lose a lot of hair during manipulation). In some cases, it may be practical to rub the cuticle of the insect with an insect pin to detach hairs, thus facilitating micropore counts. If instead of original hairiness a measure of actual hairiness is desirable, then only micropores with standing hairs should be counted.

Sometimes hairiness patterns are distinctly non‐uniform across a body part, notably in the face. In these cases, the area occupied by each hairiness pattern can be sampled separately. The percentage of surface occupied by each different density should be reported and the overall mean of hair density for that body part should be weighted by the area occupied by each hairiness pattern.

##### Hair length

The length of a hair can be measured using the length measuring tool of the software (Figure A2). Hair length is best measured from a side view. We recommend measuring 5–10 hairs in each focal body part.

##### Duration of the measurements

Following the above‐mentioned recommendations, a trained person can measure hair density and hair length of a body part in 5 min.

#### Hairiness index

We found hair length and hair density to be only weakly (and negatively) correlated. For this reason, we recommend reporting measures of the two components of hairiness (hair density and hair length) separately. However, in studies in which the two components are suspected to have a similar effect on function, we propose a hairiness index combining the two hairiness components (hair density × hair length).

#### Body size

We found that body size was positively correlated to hair length and, to a lesser extent, negatively correlated to hair density. Body size is strongly related to heat generation and dissipation and the ability to fly at low temperatures (e.g., Bishop & Armbruster, 1999; Heinrich, 1993; Osorio‐Canadas et al., 2016; Peat et al., 2005; Peters et al., 2016; Stone & Willmer, 1989), and may influence pollination effectiveness (Jauker, Speckmann, & Wolters, 2016; Kandori, 2002; Phillips et al., 2018; Willmer & Finlayson, 2014). For these reasons, we recommend accounting for body size in studies measuring hairiness. Appropriate measures of body size for bees include intertegular span (interspecific level, Cane, 1987), head width and forewing length (intraspecific level, Bosch & Vicens, 2002). Forewing length and wingspan are appropriate measures for butterflies (Beck & Kitching, 2007; García‐Barros, 2000, 2015; Miller, 1991; Nylin, Wiklund, Wickman, & Garcia‐Barros, 1993). When comparing species from different insect orders, body length is probably the most suitable measure.

#### Other considerations

We found that measuring three specimens per species allowed us to discriminate between pollinator groups and bee genera. Sample sizes should be increased in studies addressing intra‐specific variability.

Because many pollinator species (notably bees) show marked sexual dimorphism, males and females should be measured separately.

Measures of hairiness on the thorax dorsal region and body size of 109 pollinator species from Spain, Germany and Sweden are provided in Table S1.
