# Supplementary material for: A novel method to measure hairiness in bees and other insect pollinators
Source: Ecol Evol. 2020 Feb 27;10(6):2979–90. doi: 10.1002/ece3.6112 (PMC7083657; doi:10.1002/ece3.6112)
Supplement: Supplementary file 1 [file ECE3-10-2979-s001.docx]

**SUPPORTING INFORMATION**

**Tables**

Table S1. List of pollinator species and mean values of hair length and density of the dorsal surface of the mesothorax (DT), body length and intertegular span (ITS).

|  | Species name | Pollinator group | Bee taxa | DT hair length (mm) | DT hair density (hairs/mm^2^) | Body length (mm) | ITS (mm) |
| --- | --- | --- | --- | --- | --- | --- | --- |
|  |  |  |  |  |  |  |  |
| 1 | *Bombylius major* | bee-flies | - | 0.86 | 466.44 | 9.88 | - |
| 2 | *Bombylius medius* | bee-flies | - | 1.12 | 354.60 | 12.50 | - |
| 3 | *Andrena bicolor* | bees | *Andrena* | 0.59 | 256.26 | 9.50 | 2.05 |
| 4 | *Andrena cineraria* | bees | *Andrena* | 0.81 | 358.34 | 13.20 | 2.92 |
| 5 | *Andrena dorsata* | bees | *Andrena* | 0.43 | 227.65 | 11.40 | 2.09 |
| 6 | *Andrena flavipes* | bees | *Andrena* | 0.52 | 334.42 | 12.33 | 2.42 |
| 7 | *Andrena fulva* | bees | *Andrena* | 0.55 | 264.67 | 13.39 | 2.91 |
| 8 | *Andrena haemorrhoa* | bees | *Andrena* | 0.41 | 154.86 | 11.25 | 2.46 |
| 9 | *Andrena helvola* | bees | *Andrena* | 0.48 | 361.86 | 9.65 | 2.08 |
| 10 | *Andrena humilis* | bees | *Andrena* | 0.48 | 240.14 | 12.30 | 2.27 |
| 11 | *Andrena jacobi* | bees | *Andrena* | 0.70 | 154.08 | 13.45 | 2.86 |
| 12 | *Andrena lathyri* | bees | *Andrena* | 0.60 | 260.09 | 11.93 | 4.13 |
| 13 | *Andrena leptopyga* | bees | *Andrena* | 0.21 | 163.64 | 9.50 | 1.83 |
| 14 | *Andrena limata* | bees | *Andrena* | 0.66 | 501.52 | 13.51 | 2.98 |
| 15 | *Andrena minutula* | bees | *Andrena* | 0.27 | 421.03 | 7.74 | 1.54 |
| 16 | *Andrena nigroaenea* | bees | *Andrena* | 0.80 | 247.73 | 12.49 | 2.77 |
| 17 | *Andrena nitida* | bees | *Andrena* | 0.57 | 357.27 | 13.94 | 2.96 |
| 18 | *Andrena pilipes* | bees | *Andrena* | 0.62 | 159.80 | 16.30 | 3.12 |
| 19 | Andrena sp. 1 | bees | *Andrena* | 0.50 | 200.38 | 14.00 | 2.80 |
| 20 | Andrena sp.2 | bees | *Andrena* | 0.42 | 287.27 | 10.36 | 1.78 |
| 21 | *Andrena subopaca* | bees | *Andrena* | 0.24 | 408.59 | 6.39 | 1.39 |
| 22 | *Andrena vaga* | bees | *Andrena* | 0.80 | 246.33 | 14.04 | 2.78 |
| 23 | *Anthophora acervorum* | bees | Antophorinae | 0.99 | 375.69 | 14.13 | 3.98 |
| 24 | *Eucera collaris* | bees | Antophorinae | 1.07 | 135.45 | 12.53 | 3.12 |
| 25 | Eucera sp. | bees | Antophorinae | 0.92 | 157.94 | 16.50 | 3.75 |
| 26 | *Xylocopa violacea* | bees | Antophorinae | 1.00 | 63.52 | 22.40 | 5.67 |
| 27 | *Apis mellifera* | bees | *Apis* | 0.65 | 203.61 | 11.64 | 3.18 |
| 28 | *Bombus bohemicus* | bees | *Bombus* | 1.40 | 176.61 | 15.07 | 4.09 |
| 29 | *Bombus hortorum* | bees | *Bombus* | 1.58 | 301.10 | 12.83 | 3.65 |
| 30 | *Bombus humilis* | bees | *Bombus* | 1.22 | 260.55 | 12.83 | 4.26 |
| 31 | *Bombus hypnorum* | bees | *Bombus* | 1.50 | 281.03 | 13.37 | 4.22 |
| 32 | *Bombus lapidarius* | bees | *Bombus* | 1.44 | 256.77 | 14.37 | 4.50 |
| 33 | *Bombus lucorum* | bees | *Bombus* | 0.85 | 288.24 | 14.36 | 4.61 |
| 34 | *Bombus pascuorum* | bees | *Bombus* | 1.33 | 216.35 | 15.10 | 3.99 |
| 35 | *Bombus pratorum* | bees | *Bombus* | 1.05 | 345.71 | 14.41 | 3.76 |
| 36 | *Bombus sylvarum* | bees | *Bombus* | 1.22 | 253.59 | 12.77 | 3.86 |
| 37 | *Bombus terrestris* | bees | *Bombus* | 1.34 | 280.87 | 15.55 | 4.60 |
| 38 | *Halictus crenicornis* | bees | Halictini | 0.21 | 548.39 | 10.80 | 2.25 |
| 39 | *Halictus scabiosae* | bees | Halictini | 0.35 | 446.56 | 14.90 | 2.78 |
| 40 | *Halictus tumulorum* | bees | Halictini | 0.09 | 804.78 | 7.20 | 1.44 |
| 41 | *Lasioglossum calceatum* | bees | Halictini | 0.14 | 520.28 | 8.90 | 1.84 |
| 42 | *Lasioglossum fulvicorne* | bees | Halictini | 0.10 | 539.30 | 6.30 | 1.29 |
| 43 | *Lasioglossum morio* | bees | Halictini | 0.12 | 1052.24 | 5.76 | 1.14 |
| 44 | *Lasioglossum pallens* | bees | Halictini | 0.19 | 556.31 | 8.00 | 1.65 |
| 45 | *Lasioglossum pauxillum* | bees | Halictini | 0.11 | 529.03 | 6.92 | 1.32 |
| 46 | *Lasioglossum punctatissimum* | bees | Halictini | 0.10 | 681.44 | 6.10 | 1.28 |
| 47 | Lasioglossum sp. | bees | Halictini | 0.31 | 596.30 | 10.50 | 1.82 |
| 48 | *Lasioglossum zonulum* | bees | Halictini | 0.22 | 416.02 | 9.30 | 2.05 |
| 49 | *Nomada succincta* | bees | *Nomada* | 0.25 | 150.77 | 10.50 | 1.70 |
| 50 | *Osmia aurulenta* | bees | *Osmia* | 0.70 | 253.79 | 8.42 | 2.46 |
| 51 | *Osmia bicolor* | bees | *Osmia* | 0.75 | 274.03 | 9.93 | 2.72 |
| 52 | *Osmia bicornis* | bees | *Osmia* | 0.92 | 426.48 | 11.05 | 3.03 |
| 53 | *Osmia cornuta* | bees | *Osmia* | 1.17 | 363.93 | 12.38 | 3.59 |
| 54 | *Osmia tricornis* | bees | *Osmia* | 0.94 | 378.20 | 11.30 | 3.55 |
| 55 | *Agrypnus murinus* | beetles | - | 0.12 | 194.76 | 12.25 | - |
| 56 | Cantharidae sp. | beetles | - | 0.14 | 337.69 | 11.26 | - |
| 57 | *Cantharis livida* | beetles | - | 0.18 | 215.24 | 6.75 | - |
| 58 | Curculionidae | beetles | - | 0.15 | 227.89 | 5.09 | - |
| 59 | Meligethes sp. | beetles | - | 0.03 | 1875.57 | 2.40 | - |
| 60 | *Oedemera nobilis* | beetles | - | 0.16 | 313.59 | 8.60 | - |
| 61 | *Oxythyrea funesta* | beetles | - | 0.74 | 77.11 | 9.60 | - |
| 62 | Ragonycha sp. | beetles | - | 0.06 | 642.74 | 6.50 | - |
| 63 | *Tropinota squalida* | beetles | - | 0.89 | 234.68 | 11.41 | - |
| 64 | *Macroglossum stellatarum* | butterflies & moths | - | 1.34 | 616.67 | 27.92 | - |
| 65 | *Pieris brassicae* | butterflies & moths | - | 0.93 | 597.78 | 20.20 | - |
| 66 | *Pieris napi* | butterflies & moths | - | 0.97 | 951.19 | 18.67 | - |
| 67 | *Vanessa atalanta* | butterflies & moths | - | 1.91 | 1016.83 | 19.33 | - |
| 68 | *Vanessa cardui* | butterflies & moths | - | 0.58 | 636.58 | 19.75 | - |
| 69 | *Cheilosia pagana* | hover-flies | - | 0.12 | 487.23 | 6.83 | - |
| 70 | *Episyrphus balteatus* | hover-flies | - | 0.22 | 355.02 | 10.05 | - |
| 71 | *Eristalinus aeneus* | hover-flies | - | 0.35 | 373.24 | 10.84 | - |
| 72 | Eristalinus sp. | hover-flies | - | 0.43 | 283.59 | 11.75 | - |
| 73 | *Eristalis arbustorum* | hover-flies | - | 0.42 | 232.18 | 10.83 | - |
| 74 | *Eristalis interrupta* | hover-flies | - | 0.40 | 246.35 | 12.01 | - |
| 75 | *Eristalis pertinax* | hover-flies | - | 0.55 | 189.17 | 12.88 | - |
| 76 | *Eristalis similis* | hover-flies | - | 0.51 | 133.13 | 14.10 | - |
| 77 | *Eristalis tenax* | hover-flies | - | 0.63 | 234.62 | 14.28 | - |
| 78 | *Eupeodes corollae* | hover-flies | - | 0.35 | 410.79 | 8.96 | - |
| 79 | *Helophilus hybridus* | hover-flies | - | 0.35 | 365.30 | 15.00 | - |
| 80 | *Helophilus pendulus* | hover-flies | - | 0.28 | 314.91 | 12.64 | - |
| 81 | *Helophilus trivitattus* | hover-flies | - | 0.38 | 458.84 | 16.17 | - |
| 82 | *Melanostoma mellinum* | hover-flies | - | 0.13 | 653.82 | 7.55 | - |
| 83 | *Melanostoma scalare* | hover-flies | - | 0.20 | 443.66 | 7.63 | - |
| 84 | *Meliscaeva auricollis* | hover-flies | - | 0.17 | 269.85 | 8.50 | - |
| 85 | *Myathropa florea* | hover-flies | - | 0.44 | 232.22 | 11.80 | - |
| 86 | *Neoascia podagrica* | hover-flies | - | 0.05 | 907.95 | 5.60 | - |
| 87 | *Platycheirus albimanus* | hover-flies | - | 0.07 | 502.20 | 7.90 | - |
| 88 | *Platycheirus peltatus* | hover-flies | - | 0.16 | 425.95 | 8.85 | - |
| 89 | *Rhingia campestris* | hover-flies | - | 0.32 | 399.42 | 8.67 | - |
| 90 | *Scaeva albomaculata* | hover-flies | - | 0.43 | 404.00 | 13.00 | - |
| 91 | *Scaeva pyrastri* | hover-flies | - | 0.39 | 234.47 | 12.83 | - |
| 92 | *Sphaerophoria scripta* | hover-flies | - | 0.13 | 338.73 | 9.00 | - |
| 93 | *Syrphus ribesii* | hover-flies | - | 0.33 | 314.70 | 11.40 | - |
| 94 | *Syrphus vitripennis* | hover-flies | - | 0.32 | 291.70 | 9.90 | - |
| 95 | *Xanthandrus comtus* | hover-flies | - | 0.19 | 341.78 | 10.78 | - |
| 96 | Anthomyiidae | other flies | - | 0.30 | 85.93 | 4.32 | - |
| 97 | *Bibio hortulanus* | other flies | - | 0.14 | 192.90 | 11.00 | - |
| 98 | Dilophus sp. | other flies | - | 0.31 | 228.95 | 5.33 | - |
| 99 | Empis sp. | other flies | - | 0.19 | 199.78 | 10.20 | - |
| 100 | Large fly | other flies | - | 0.38 | 416.65 | 10.50 | - |
| 101 | Medium-sized fly | other flies | - | 0.28 | 315.52 | 7.75 | - |
| 102 | *Neomyia cornicina* | other flies | - | 0.24 | 149.06 | 6.90 | - |
| 103 | *Sarcophaga carnaria* | other flies | - | 0.30 | 73.93 | 10.50 | - |
| 104 | Small fly | other flies | - | 0.40 | 84.71 | 5.00 | - |
| 105 | *Hoplocampa testudinea* | saw-flies | - | 0.04 | 1027.34 | 6.17 | - |
| 106 | *Tenthredo koehleri* | saw-flies | - | 0.10 | 480.56 | 9.00 | - |
| 107 | *Polistes dominulus* | wasps | - | 0.07 | 1444.35 | 14.50 | - |
| 108 | *Vespula germanica* | wasps | - | 0.58 | 145.45 | 14.83 | - |
| 109 | *Vespula vulgaris* | wasps | - | 0.89 | 116.63 | 20.00 | - |

Table S2. Correlations (Pearson *r* or Spearman *ρ*) of body size (body length (BL) for all pollinators, intertegular span (ITS) for bees) with hair density and hair length in each body part (dorsal thorax: DT, ventral thorax: VT; face: FA).

| **All pollinators** |  |  | **Bees** |  |  |
| --- | --- | --- | --- | --- | --- |
| BL^a^ | **Body Part** | **r/ρ** | ITS^a^ | **Body Part** | **r/ρ** |
|  | DT^a^ | r = -0.20* |  | DT^a^ | r = -0.55*** |
| Hair density | VT | ρ = 0.06 n.s. | Hair density | VT^a^ | r = -0.50*** |
|  | FA | ρ = 0.12 n.s. |  | FA^a^ | r = -0.48*** |
|  | DT^a^ | r = 0.67*** |  | DT^a^ | r = 0.89*** |
| Hair length | VT^b^ | r = 0.70*** | Hair length | VT^b^ | r = 0.93*** |
|  | FA^b^ | r = 0.70*** |  | FA^b^ | r = 0.91*** |

n.s.: non-significant; *: P ≤ 0.05;** : P ≤ 0.01 and *** : P ≤ 0.001

Data transformations: ^a^ Log (X + 1) and ^b^ Square root.

Table S3. Correlation coefficients (Pearson *r* and Spearman *ρ*) of hair density, hair length and hairiness index among body parts (dorsal thorax: DT, ventral thorax: VT; face: FA).

| All pollinators | | Bees | |  |
| --- | --- | --- | --- | --- |
| Body part (A-B) | r/ρ | Body part (A-B) | r/ρ |  |
| Hair density | DT - VT | ρ = 0.47*** | DT^a^ - VT^a^ | r = 0.68*** |
|  | DT - FA | ρ = 0.48*** | DT^a^ - FA^a^ | r = 0.61*** |
|  | VT - FA | ρ = 0.67*** | VT^a^ - FA^a^ | r = 0.74*** |
| Hair length | DT^a^ - VT^b^ | r = 0.95*** | DT^b^ - VT^b^ | r = 0.96*** |
|  | DT^a^ - FA^b^ | r = 0.90*** | DT^b^ - FA^b^ | r = 0.96*** |
|  | VT^b^ - FA^b^ | r = 0.91*** | VT^b^ - FA^b^ | r = 0.96*** |
| Hairiness index | DT^a^ - VT^a^ | r = 0.80*** | DT^a^ - VT^a^ | r = 0.80*** |
|  | DT^a^ - FA^a^ | r = 0.78*** | DT^a^ - FA^a^ | r = 0.77*** |
|  | VT^a^ - FA^a^ | r = 0.88*** | VT^a^ - FA^a^ | r = 0.85*** |

*: P ≤ 0.05;** : P ≤ 0.01 and *** : P ≤ 0.001

Data transformations: ^a^ Log (X + 1) and ^b^ Square root.

Table S4. Within and between species coefficients of variation (CV, %) of hair density and hair length of three body parts (dorsal thorax: DT, ventral thorax: VT; face: FA) for all pollinators and for bees.

|  |  | **All pollinators** | | **Bees** | |
| --- | --- | --- | --- | --- | --- |
|  | **Body Part** | **CV within** | **CV between** | **CV within** | **CV between** |
| Hair density | DT | 18.7 | 73.0 | 18.3 | 53.4 |
| Hair length |  | 17.1 | 76.7 | 17.0 | 60.7 |
| Hair density | VT | 23.6 | 103.0 | 21.7 | 38.3 |
| Hair length |  | 18.5 | 65.9 | 17.0 | 48.5 |
| Hair density | FA | 22.1 | 145.3 | 20.0 | 38.6 |
| Hair length |  | 18.3 | 67.9 | 17.0 | 50.3 |

Table S5. Statistical outputs of ANOVA (F-value) and Kruskal Wallis (H-statistic) tests analyzing differences in hair length, hair density and hairiness index between pollinator (n=109 species) and bee groups (n=52 species) of three body parts (dorsal thorax: DT, ventral thorax: VT; face: FA).

|  |  | DT | | VT | | | FA | | |
| --- | --- | --- | --- | --- | --- | --- | --- | --- | --- |
|  | DF |  | F |  | F/H |  |  | F/H |  |
| All pollinators | 4 | Hair density^a^ | 6.92*** | Hair density | H | 32.50*** | Hair density | H | 44.68*** |
|  | 4 | Hair length^a^ | 11.34*** | Hair length^b^ | F | 17.14*** | Hair length^b^ | F | 16.08*** |
|  | 4 | Hairiness index^a^ | 27.21*** | Hairiness index^a^ | F | 39.87*** | Hairiness index^a^ | F | 15.89*** |
|  | 4 | Hair density^a^ | 16.25*** | Hair density^a^ | F | 4.06** | Hair density^a^ | F | 2.62* |
| Bees | 4 | Hair length^a^ | 67.90*** | Hair length^b^ | F | 40.09*** | Hair length^a^ | F | 54.20*** |
|  | 4 | Hairiness index^a^ | 13.52*** | Hairiness index^a^ | F | 22.53*** | Hairiness index^a^ | F | 19.96*** |

*: P ≤ 0.05;** : P ≤ 0.01 and *** : P ≤ 0.001

Data transformations: ^a^ Log (X + 1) and ^b^ Square root.

**Figures**


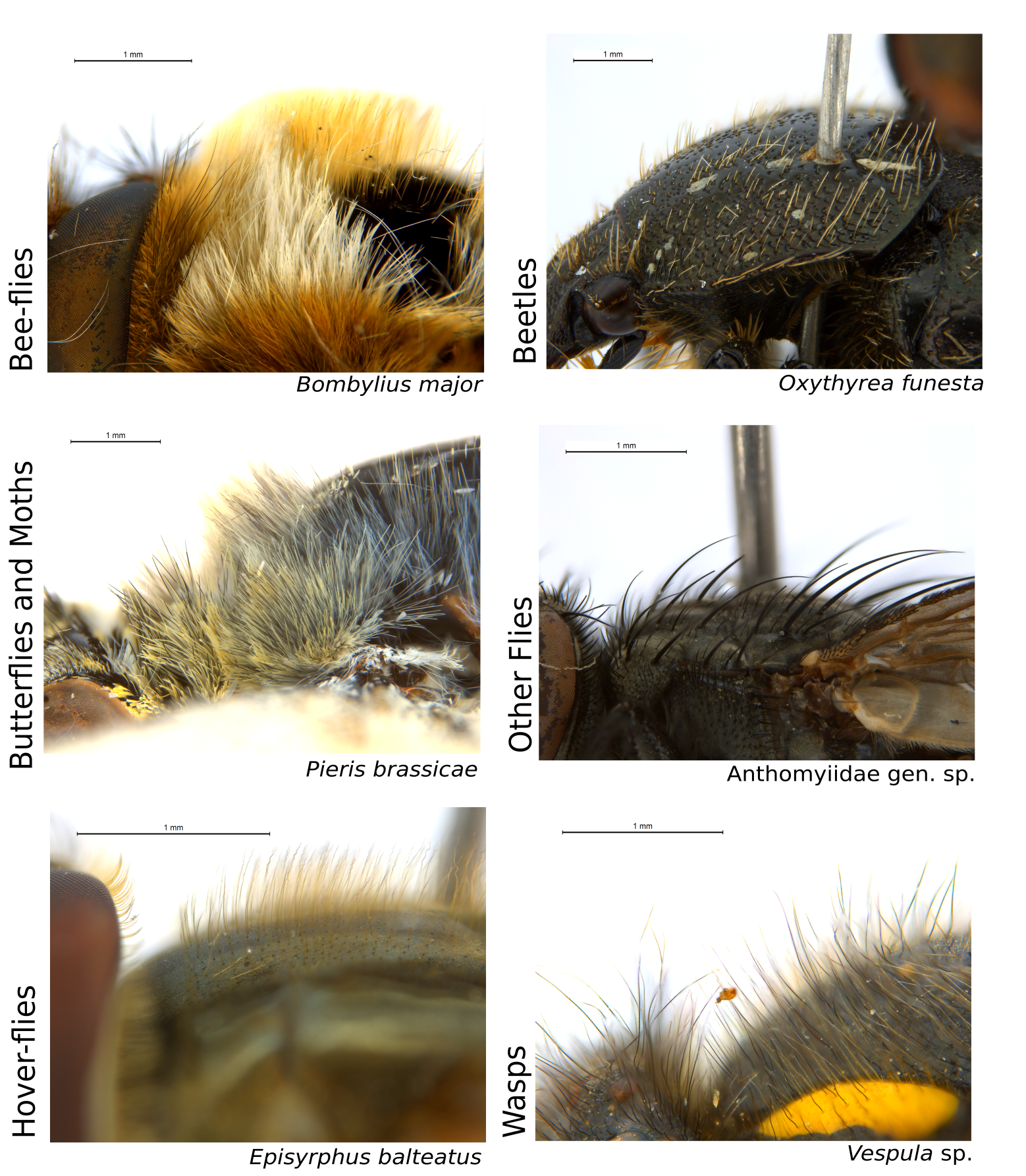


Figure S1. Lateral view of hairs from the dorsal surface of the mesothorax of six non-bee pollinators.


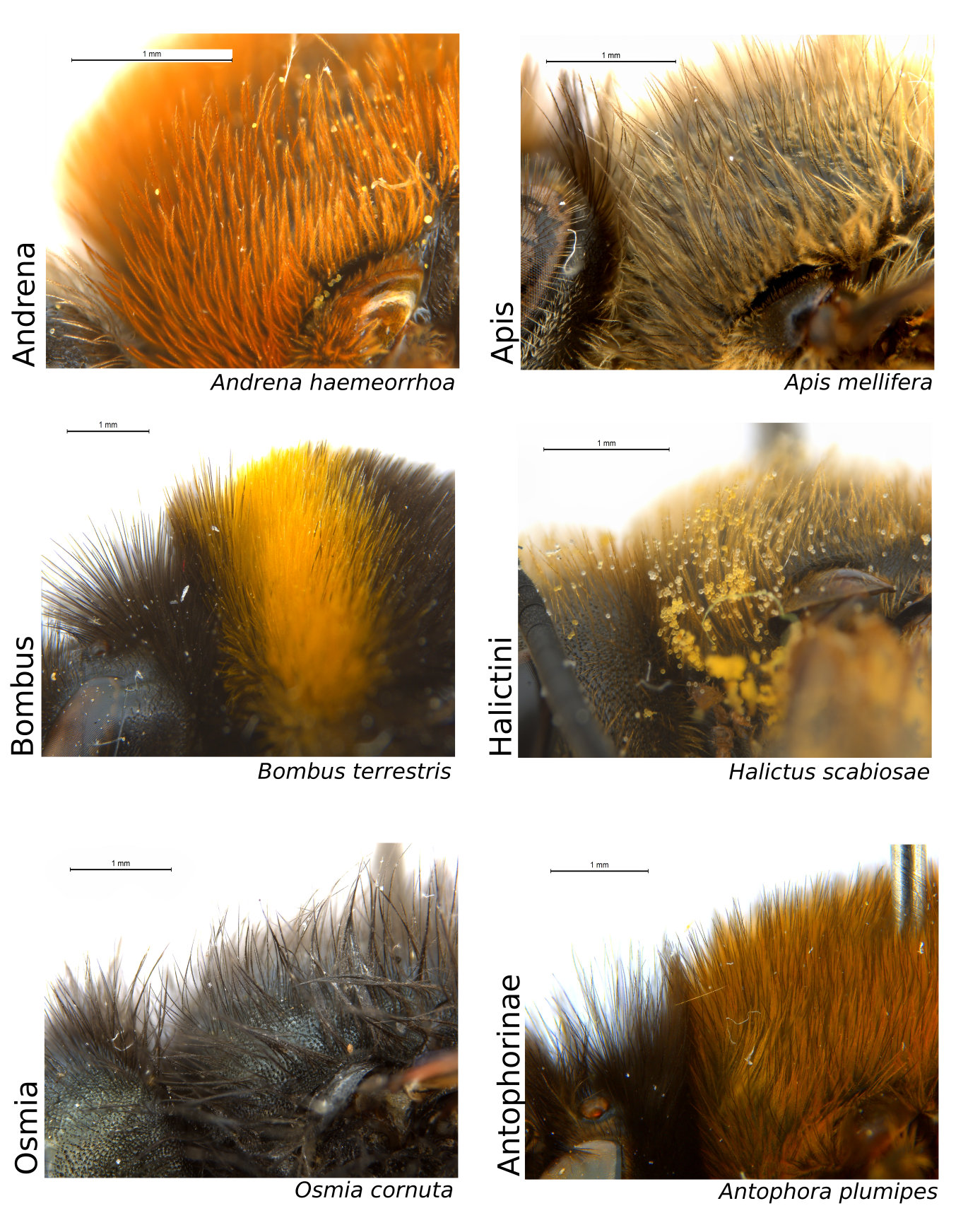


Figure S2. Lateral view of hairs from the dorsal surface of the mesothorax of six bee species.


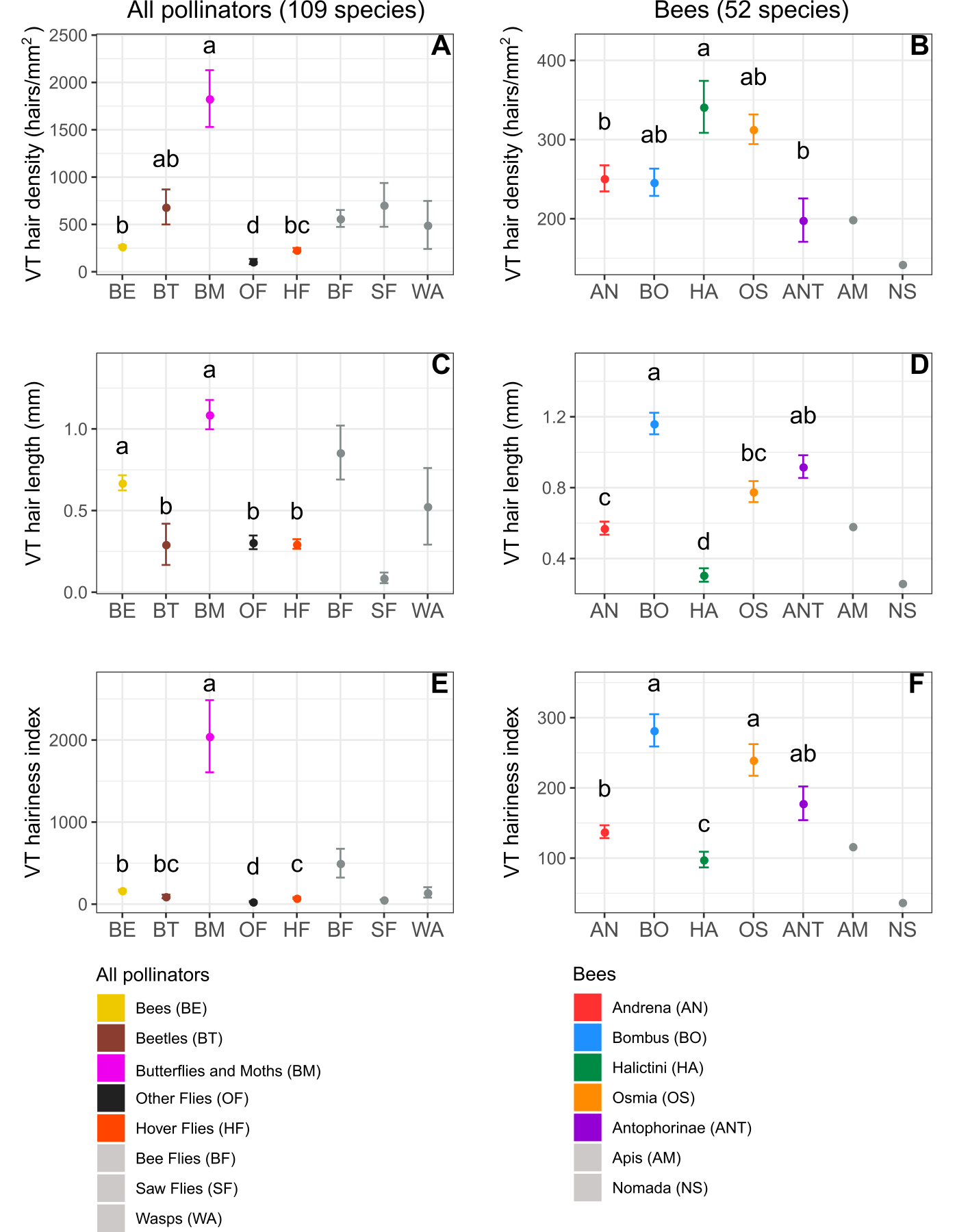


Figure S3. Mean ± SE hair density (A, B), hair length (C, D) and hairiness index (E, F) of the ventral surface of the mesothorax (VT) of various pollinator groups and bee taxa. Different letters indicate significant differences among groups (post-hoc Tukey tests, P < 0.05). Grey bars correspond to groups with fewer than three species that were not included in the analyses.


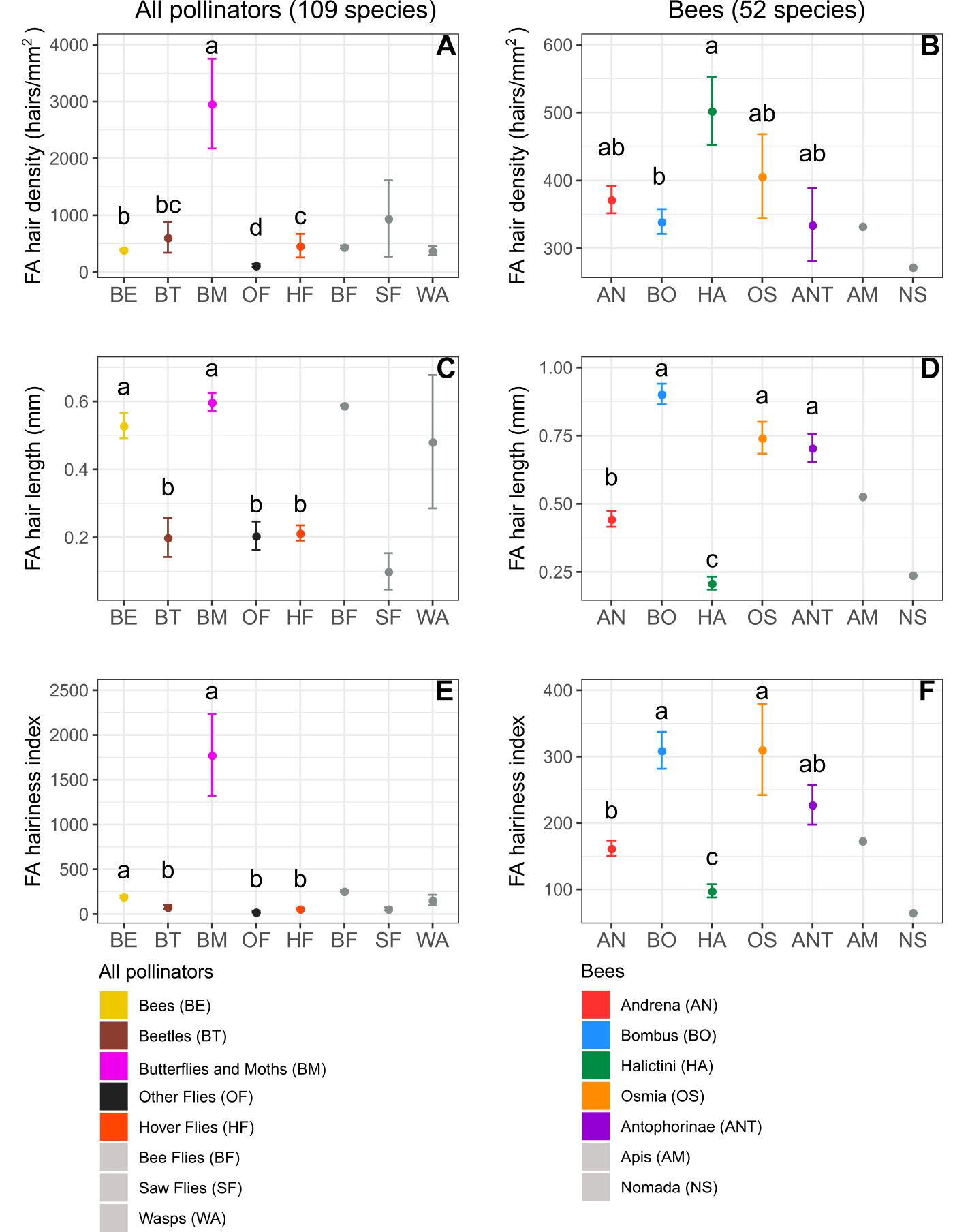


Figure S4. Mean ± SE hair density (A, B), hair length (C, D) and hairiness index (E, F) of the face (FA) of various pollinator groups and bee taxa. Different letters indicate significant differences among groups (post-hoc Tukey tests, P < 0.05). Grey bars correspond to groups with fewer than three species that were not included in the analyses.
